# The Assembly of Tropical Dry Forest Tree Communities in Anthropogenic Landscapes: The Role of Chemical Defenses

**DOI:** 10.3390/plants11040516

**Published:** 2022-02-14

**Authors:** Ángel E. Bravo-Monzón, Cristina Montiel-González, Julieta Benítez-Malvido, María Leticia Arena-Ortíz, José Israel Flores-Puerto, Xavier Chiappa-Carrara, Luis Daniel Avila-Cabadilla, Mariana Yolotl Alvarez-Añorve

**Affiliations:** 1 Laboratorio de Ecología Funcional de Ecosistemas Terrestres, Escuela Nacional de Estudios Superiores Unidad Mérida, Universidad Nacional Autónoma de México, Mérida 97357, Yucatán, Mexico; abravomonzon@gmail.com (Á.E.B.-M.); montigcris@gmail.com (C.M.-G.); Kanan-IFP@outlook.com (J.I.F.-P.); 2 Laboratorio de Ecología de Hábitats Alterados, Instituto de Investigaciones en Ecosistemas y Sustentabilidad, Universidad Nacional Autónoma de México, Morelia 58190, Michoacán, Mexico; jbenitez@cieco.unam.mx; 3 Laboratorio de Ecogenómica, Facultad de Ciencias, Universidad Nacional Autónoma de México, Parque Científico y Tecnológico, Mérida 97302, Yucatán, Mexico; leticia.arena@ciencias.unam.mx; 4 Escuela Nacional de Estudios Superiores Unidad Mérida, Universidad Nacional Autónoma de México, Mérida 97357, Yucatán, Mexico; xavier@enesmerida.unam.mx

**Keywords:** tropical dry forest, community assembly, leaf traits, secondary metabolites

## Abstract

The effect of anthropogenic disturbance on plant community traits and tradeoffs remains poorly explored in tropical forests. In this study, we aimed to identify tradeoffs between defense and other plant functions related to growth processes in order to detect potential aboveground and edaphic environmental conditions modulating traits variation on plant communities, and to find potential assembly rules underlying species coexistence in secondary (SEF) and old-growth forests (OGF). We measured the foliar content of defense phytochemicals and leaf traits related to fundamental functions on 77 species found in SEF and OGF sites in the Jalisco dry forest ecoregion, Mexico, and we explored (1) the trait-trait and trait-habitat associations, (2) the intra and interspecies trait variation, and (3) the traits-environment associations. We found that phytochemical content was associated with high leaf density and leaf fresh mass, resulting in leaves resistant to drought and high radiation, with chemical and physical defenses against herbivore/pathogen attack. The phytochemicals and chlorophyll concentrations were negatively related, matching the predictions of the Protein Competition Model. The phylogenetic signal in functional traits, suggests that abundant clades share the ability to resist the harsh biotic and abiotic conditions and face similar tradeoffs between productive and defensive functions. Environmental filters could modulate the enhanced expression of defensive phytochemicals in SEF, while, in OGFs, we found a stronger filtering effect driving community assembly. This could allow for the coexistence of different defensive strategies in OGFs, where a greater species richness could dilute the prevalence of pathogens/herbivores. Consequently, anthropogenic disturbance could alter TDF ecosystem properties/services and functioning.

## 1. Introduction

Studying the variation in plant functional traits, such as the ones related to water use, light acquisition, heat regulation, and defense against herbivores and pathogens, has proven useful for tackling many important ecological questions [1,2]. Within communities, trait variation is the result of multiple biotic and abiotic forces that interact dynamically to drive the community pattern. The strength and direction of biotic interactions can be strongly influenced by the abiotic context and abiotic events throughout the life span of an organism and thus can have strong influences on community membership [3,4,5]. 

Specifically, the study of foliar chemical traits with defensive value is particularly important in tropical forests with high and increasing herbivore and pathogen pressure as a result of fragmentation, invasive species, and anthropogenic disturbance [6,7,8]. Nevertheless, challenges associated with quantifying phytochemical traits at the community level have limited our understanding of the causes and consequences of variation in phytochemical diversity across plant species [9]. As large areas of tropical forests are being transformed by human activities, it is essential to characterize the functional variation in conserved and disturbed tropical plant communities to understand how habitat alteration could affect the functional performance of the vegetation as well as its susceptibility to the attack of herbivores and pathogens. Additionally, because variation in plant traits is modulated by biotic and abiotic factors that also regulate plant diversity [10,11], the aforementioned traits could be indicative of current assembly rules (i.e., rules that explain the assemblage and abundance of species in a community) in tropical communities. 

A useful framework for understanding how plants will balance their allocation of resources under different environmental conditions is the Growth-Differentiation Balance (GDB) Hypothesis [12,13]. This hypothesis makes a distinction between growth processes (i.e., those that require cellular division or elongation to produce new stems, roots, leaves, etc.) and differentiation processes (i.e., those that improve the structure or function of existing cells). Secondary metabolites and thick leaves are examples of differentiation processes that, while reducing herbivory, are considered to compete for plant resources [14,15,16]. The GDB hypothesis proposes three scenarios, depending on the available resources: (1) plants growing under low resources will be limited in their growth and photosynthetic capacity; (2) plants in an intermediate level of resources should have high concentrations of secondary metabolites but intermediate biomass accumulation; (3) plants in high resources should not be limited in their growth or photosynthetic capacity [17]. So far, the GDB hypothesis has received only partial support [18,19].

With this study, we pursued the following objectives: (a) to characterize plant strategies in a tropical dry forest and to identify potential tradeoffs between defense—a differentiation process—and other plant functions at the leaf level related to growth processes, (b) to compare old-growth forest (OGF) and secondary forest (SEF) tree communities in terms of plant defense strategies and to find potential assembly rules underlying species coexistence in the two types of forests, and (c) to detect potential aboveground and edaphic environmental conditions modulating traits variation on plant communities. For this, we tested the following hypotheses: (1) the content of secondary metabolites (i.e., total phenols, tannins, and flavonoids), having functions of photoprotection and chemical defense of leaf tissues, will be related to plant functional traits enhancing light acquisition and related, in general, to plant growth. In this regard, we predict that a tradeoff between production and defense could be occurring. (2) The different biotic (abundance of herbivores/pathogens) and abiotic conditions (radiation, temperature) imposed by the successional stage will reflect in the content variation of phytochemicals between communities; we expect a higher phytochemical content in disturbed forests because radiation and abundance of herbivores/pathogens tend to be higher in tropical secondary forest [20,21,22]. (3) The community assemblage will be determined by interacting internal and external filters (i.e., biotic and abiotic factors). In this regard, we expect to find a predominance of internal filters in the OGF, where the most specious communities, with a high competition intensity among species, occur. We also expect a greater prevalence of external filters in SEF, where high solar radiation, temperature, and low water availability could be limiting the species’ community membership. In the end, we expect that the tradeoffs among plant species dealing with the particular combination of resources, environmental conditions (i.e., environmental stresses), and biotic pressures (i.e., herbivores, parasites, and pathogens) prevailing in disturbed tropical forests, can explain changes in plant community functional composition in anthropogenic landscapes.

To reach our objectives, we analyzed and compared the foliar functional traits of tree communities from two habitat types (OGF vs. SEF communities), in a tropical dry forest region (TDF), and looked for potential tradeoffs between defense and other functional traits related to plant development and growth. We also evaluated potential assembly rules explaining species coexistence in this tropical system and explored how such rules could be changing among successional stages by considering not only the interspecific trait variability but also the intraspecific trait variability, which can have significant effects on community dynamics and ecosystem functioning [23,24,25]. The approach we used to identify assembly rules proposes a categorization of the assembly processes into (i) external filters, those that operate at spatial scales larger than the community (i.e., climate, soil, generalist predators/pathogens), and (ii) internal filters, those occurring within the community (i.e., competition, parasitism, microenvironmental variability) [25].

## 2. Materials and Methods

### 2.1. Study Site 

This study was carried out in and around the Chamela-Cuixmala Biosphere Reserve (19°22′–19°35′ N, 104°56′–105°03′ W) which is located in the western coast of Mexico—Jalisco dry forest ecoregion [26]. This tropical region is characterized by a seasonal fluctuation in the precipitation regime—the dry season lasts approximately seven months—which determines the tropical dry forest (TDF) as the predominant natural vegetation [27]. However, during the last 40 years, TDF in the Chamela region has suffered a reduction in its coverage of approximately 22%, mainly due to agriculture, cattle raising, and, to a lesser degree, urban expansion [28].

The vegetation sampling for this study was carried out in a set of permanent sites established to study the functionality and diversity of plant communities in tropical dry forests [29]. Specifically, we used eight sampling sites representing two habitat types, four old-growth forests (OGF) sites (LIM2, TEJ1, TEJ2, and UNAM), and four secondary forests (SEF) sites (HN2, LFH, LIM2, and NAC2). The sites were selected with the help of high-resolution imagery (i.e., satellite imagery in Google Earth; http://earth.google.com (accessed on 2 April 2015)), land cover maps based on satellite aerial imagery, and data collected through interviews with the landowners. On each site, we established a standardized rectangular 0.1-ha plot (50 × 20 m), including a homogeneous piece of forest. When selecting the sites, we seek to correct for the differences between them in terms of their elevation, slope, and aspect, which are tightly related to variation in environmental conditions and resources availability (i.e., radiation and humidity), in order to focus the analysis on the differences between the two types of habitats considered. As a result, the set of sites selected for the study were located at an average mean elevation of 143 m.a.s.l, with slopes averaging 10° and mostly facing toward the south and the southwest. The criteria for establishing the study sites are described in detail in Jimenez-Rodríguez et al. [29]. 

### 2.2. Vegetation Sampling and Plant Functional Traits Measurements

On each sampling plot, we identified all woody species with a diameter at breast height (DBH; 1.30 m height) equal to or greater than 2.5 cm. After that, we collected five healthy, fully expanded, sun-exposed, mature leaves from the most abundant species representing at least 70% of total woody individuals in each site, which are those that potentially have a greater impact on the ecological processes [30]. 

The collected leaves were processed following the next steps: (1) they were read with the SPAD 502 Plus Meter (Konica Minolta Sensing, Europe) to estimate chlorophyll content (CC); (2) they were marked and placed in sealed plastic bags containing moistened paper towels and transported in a cooler to the laboratory; and (3) they were processed in the lab following standard methods [2,29] to measure specific leaf area (SLA, leaf area per dry mass), leaf density (LD, leaf dry mass/(leaf area × leaf thickness)) and leaf fresh mass per unit area (LFM).

#### Quantification of Total Phenols, Tannins, and Flavonoids

For each sampled individual, we mixed and processed three oven-dried leaves (70 °C × 72 h) for the quantification of total phenols (including total tannins, and total flavonoids) because the GDB, a resource-based theory, can only make valid predictions regarding the total amount of carbon allocated to secondary compounds and fails at making predictions on the allocation of resources at finer hierarchical classes [31]. For this purpose, we separated a 50 mg sample of foliar tissue, which was homogenized using an ice-cold mortar and pestle, and 2 mL of cold 95% methanol. The sample was placed in a 2 mL microtube and incubated at room temperature for 48 h in the dark. Afterward, samples were centrifuged (5000× *g* for 4 min at room temperature) and the methanolic supernatant was collected in a new 2 mL microtube. Secondary metabolites were then quantified using colorimetric methods and a multiplate spectrophotometer (Thermo Scientific Multiskan GO).

Specifically, for the total phenol quantification, we used a modified version of the Folin–Ciocalteu colorimetric assay developed for microcentrifuge tubes and plate readers [32] to quantify total phenols (TP) [33]. We added 40 μL of supernatant to 960 μL of 95% methanol in a microtube and vortexed for 10 s (Appendix A). A 100 μL aliquot was placed in a new tube with 200 μL of Folin–Ciocalteu reagent (10%), vortexed for 10 s, and incubated for 30 min at room temperature. After, 800 μL of sodium carbonate (700 mM) were added, vortexed for 10 s and incubated for 2 h at room temperature. We centrifuged samples for 10 min at 5000 rpm and transferred 300 μL of each sample into 96 well microplates. A blank with no supernatant was prepared for each microplate. Absorbance was measured at 735 nm. A standard curve was prepared using gallic acid in concentrations from 25 to 200 mg/L. Total phenol concentration was expressed as mg gallic acid equivalents (GAE)/100 g dry mass.

For the analysis of total tannins, we used a modified version of a previously reported method [34] to quantify tannins (T) [33]. The remaining 900 μL aliquot from the phenol analysis was transferred to a new microtube containing 20 mg of polyvinylpyrrolidone (PVP), vortexed for 10 s, and incubated for 30 min (Appendix A). Absorbance was measured at 735 nm. Tannins concentrations were calculated using the regression formula from a gallic acid standards curve with concentrations from 25 to 200 mg/L. Total tannin concentration was expressed as mg gallic acid equivalents (GAE)/100 g dry mass.

Finally, we used the aluminum chloride colorimetric assay [35] to quantify flavonoids (F). We added 20 μL of methanolic supernatant to a microtube containing 80 μL of dd H_2_O (distilled deionized water) and vortexed for 10 s (Appendix A). After, 30 μL of NaNO_2_ (5%) were added and let stand for 5 min before adding 30 μL of AlCl_3_ (10%). After 1 min, we added 200 μL of NaOH (1M) and the volume was brought to 1 mL with dd H_2_O. The mixture was vortexed for 10 s, and 300 μL were transferred to a microplate to measure its absorbance at 510 nm [33]. A standard curve was prepared using (+)-catechin in concentrations from 20 to 200 mg/L. Total flavonoid content was expressed as mg catechin equivalents (CE)/100 g dry mass.

### 2.3. Environmental Characterization

#### 2.3.1. Environmental and Biogeochemical Soil Factors Analyses

A total of five soil samples per site (one at the center and one at each corner) were taken with a soil-core sampler from the first 15 cm of soil depth, after removal of any litter layer. These samples were later combined to produce one composite sample per site (eight composite samples in total) and stored in black plastic bags at 4 °C until laboratory analysis.

Two environmental soil variables were measured: pH and soil humidity. The pH was measured in deionized water (soil/solution, 1:2, *w*:*v*) with a digital pH meter (Corning TM). For the soil humidity determination, from each sample, a subsample of 100 g was oven dried at 75 °C to constant weight for soil moisture determination using the gravimetric method [36].

For the determination of total nutrients, 50 g of fresh soil were dried and milled with a pestle and mortar. The total carbon (TC) was measured from a processed subsample of 15 mg. To determine the total nitrogen (TN) and phosphorus (TPho), subsamples of 5 g were digested in a mixture of concentrated H_2_SO_4_, H_2_O_2_ (30%), and K_2_SO_4_ plus CuSO_4_, the latter acting as a catalyst at 360 °C. One aliquot of the extract was used to determine the TPho and TN.

The dissolved inorganic nitrogen forms (ammonium DNH_4_^+^ and nitrate DNO_3_^−^) and phosphorus (phosphate, PO_4_^+^) were extracted from 20 g of fresh field soil samples with deionized water after shaking for 45 min and then filtering through a Whatman filter paper No. 42 and a 0.45 nitrocellulose membrane. One aliquot of the filtrate was used to determine the dissolved nitrogen and phosphorus [37].

The Total Organic Carbon (TOC) was determined by combustion and coulometric detection [38] with a Total Carbon Analyzer (UIC Mod. CM5012; Chicago, IL, USA). The nitrogen (N) forms were determined by the macro Kjeldahl method [39] and the phosphorus (Pho) was determined by the molybdate colorimetric method, following ascorbic acid reduction [40]. Both N and P forms were measured with colorimetrical analyses using a Bran Luebbe Auto Analyzer III (Norderstedt, Germany). 

The activities of three enzymes (extracellular) involved in the cleavage of organic molecules with C, N, and P were measured: β-1, 4-glucosidase (BG), β-1, 4-N-acetylglucosaminidase (NAG), and phosphomonoesterase (PME) with the assay techniques reported by Tabatabai and Bremner [41], Eivazi and Tabatabai [42], Eivazi and Tabatabai [43] and Verchot and Borelli [44]. For all enzymes, we used 2 g of fresh soil and 30 mL of modified universal buffer (MUB) at pH 9 for enzyme extraction. Three replicates and two control samples (soil extract with no substrate, and pure MUB with substrate) were included per assay. All enzyme assays were incubated at 40 °C, the BG for 2 h, the NAG for 3 h, and the PME for 1.25 h. Following the incubation period, the tubes were centrifuged at 10,000 rpm for 2 min and 750 μL of supernatant were recovered. We diluted the supernatant in 2 mL of deionized water with 75 μL of NaOH and measured the absorbance of p-nitrophenol (pNP) liberated at 410 nm on an Evolution 201 spectrophotometer (Thermo Scientific Inc., Mexico City, Mexico). Enzyme activities were expressed as nanomoles of pNP per gram of dry soil per hour (nmol pNP [g SDE]^−1^ h^−1^).

#### 2.3.2. Environmental Data Collection

During the leaf sampling period, we also measured environmental parameters in each study site using a HOBO U30 station data logger (ONSET, Bourne, MA, USA), which monitored continuously (every 10 s) and simultaneously six environmental variables during 3 days: (1) the environmental/air average temperature (ET_avg_), with the RH Smart Sensor S-TBH-M00x; (2) the environmental/air average humidity (EH_avg_), with the sensor S-TBH-M00x; (3) the maximum sun radiation (SR_max_), with the Silicon Pyranometer Smart Sensor S-LIB-M003); (4) the average of photosynthetically active radiation (PAR_avg_), with the PAR Smart Sensor S-LIA-M003; (5) the average soil temperature (ST_avg_), with the 12-Bit Temperature Smart Sensor S-TMB-M0xx; and (6) soil water content (SWC), with the Soil Moisture Smart Sensor S-SMx-M005. The aerial sensors were always located above the understory layer, while the ground sensors were located at approximately 10 cm of soil depth, below the litter layer. 

All environmental parameters were measured in the middle of the rainy season. These parameters significantly respond to the variation in vegetation structure (i.e., with an increase in vegetation structural complexity, temperature and radiation decrease while the relative humidity increase), which in turn can affect the plant functional performance [22]. All the parameters were evaluated for their potential effect on leaf traits (see data analysis section).

### 2.4. Data Analysis

#### 2.4.1. Relationships between Leaf Defense Traits and Plant Functional Traits

First, we tested for the phylogenetic signal of each trait by contrasting the results obtained with different approaches: (1) methods based on the autocorrelation principle (i.e., the Moran’s and the Abouheif’s C_mean_ index); and (2) methods based on evolutionary models (i.e., Blomberg’s K, K* and Pagel’s λ). We contrasted these methods to identify robust patterns in traits evolution because the results of every single index can strongly depend on (I) the phylogenetic tree topology; (II) the sample size; and (III) the complexity of the evolutionary model generating traits patterns [45]. We built the phylogenetic tree including all the studied species, using as a backbone the megaphylogeny (time-calibrated tree) provided by Zanne et al. [46] and updated by Qian and Jin [47], which includes 98.6% and 51.6% of all extant seed plant families and genera, respectively. In the end, we generated an ultrametric tree with branch lengths in units of time (millions of years), considering the taxonomic designation of “World Flora Online” (www.worldfloraonline.org (accessed on 1 June 2020)). 

To characterize defensive strategies across the species and to determine potential tradeoffs between plant traits related to leaf defense, water use, light acquisition, and heat load regulation, we quantified the degree of relationship between traits using the method of Zheng et al. (2009) [48], which allowed us to quantify the covariance between a pair of traits while accounting for (1) the correlation between traits that is fixed across the tree; (2) the standard error associated with the estimation of each trait; and (3) the phylogenetic signal associated to each trait [48]. Finally, we calculated the Spearman correlation coefficient between traits and tested its significance (*p* < 0.05) considering the individual’s trait variation for the most abundant species—those accounting for 70% of the overall sampled individuals.

#### 2.4.2. Contrasting OGF and SEF Plant Communities in Terms of Their Functional Composition

We used non-Metric Multidimensional Scaling (NMDS), an unconstrained ordination method, to map the overall variation among plant communities in terms of their functional traits, and to detect significant differences among plant communities from different sites and habitat types. This analysis was carried out considering the variation of the functional traits at the individual level in each community. For this analysis, we followed the next steps: (1) we normalized the datasets; (2) we built a Euclidian distance matrix based on such datasets; (3) we mapped, with the help of the NMDS algorithm, the individuals into a multidimensional functional space, to visualize the dissimilarities among them; (4) we estimated the centroids corresponding to each study site and habitat type in the multidimensional space; and (5) we evaluated if the observed distances among centroids regarding habitats and sites were greater than expected by chance (*p* < 0.05 for significance testing), employing Monte Carlo tests based on 999 permutations. We used the stress value (scaled from 0 to 100) to evaluate how successfully the ordinations graphically represent the dissimilarities between individuals, where low stress values indicate more reliable ordinations. Finally, we calculated the community-weighted mean (CWM) regarding every trait, as a measure of functional composition, and contrasted them among types of forests using analysis of variance (ANOVA). The CWM was calculated using the following formula: CWM = (W_1_ × X_1_) (W_2_ × X_2_) ... (W_i_ × X_i_) (W_n_ × X_n_), where n = number of species, W = relative abundance of the ith species, and X = the average of the attribute value of the ith species. 

#### 2.4.3. Evaluation of Potential Assembly Rules Underlying Species Coexistence in OGF and SEF Sites

We calculated the series of three T-statistics (T*_IP/IC_*, T*_IC/IR_*, and T*_PC/PR_*) proposed by Violle et al. [25] for each functional trait. These statistics quantify the strength of internal filtering (T*_IP/IC_*), and external filtering operating on individuals’ values (T*_IC/IR_*) and species averages (T*_PC/PR_*). The calculus of the T-statistics is based on the ratio of different components of phenotypic traits variation across spatial scales (i.e., at vegetation stand and regional levels) and biological levels (i.e., at population and community level). Specifically, the estimated components of phenotypic traits variation for the T-statistics calculations were the variance among individuals within populations (σ^2^_IP_), within communities (σ^2^_IC_), and within the regional pool (σ^2^_IR_); the variance of population mean traits values within communities (σ^2^_PC_), and within the regional pool (σ^2^_PR_). Then, we calculated the three T-statistics as follows: (1) T*_IP/IC_* = σ^2^_IP_/σ^2^_IC_, being a measure of interspecific niche packing in the communities—low values indicate a reduction of local intraspecific variations while high values indicate strong niche overlapping; (2) T*_IC/IR_* = σ^2^_IC_/σ^2^_IR_, being a measure of changes in the variance within a community relative to the total variance of the regional pool, regardless of the species identity—individuals are considered together regardless of the species to which they belong, where values close to 0 could be indicative of the action of external filtering operating at the individual level; and (3) T*_PC/PR_* = σ^2^_PC_/σ^2^_PR_, being a measure of changes in the variance among species within a community relative to the total variance among the species conforming the regional pool, where values close to 0 could be indicative of the action of filtering operating at the population level. The significant deviation (alpha = 0.05) of each statistic from random expectations was evaluated by performing specific null model tests, based on 999 randomizations, for each T-statistic: (1) for T*_IP/IC_*, individual traits values are permuted within communities, preserving the species composition unchanged (null model = “local”); (2) for T*_IC/IR_*, traits values are permuted for all individuals in all communities, preserving the number of individuals in each community unchanged (null model = “regional.ind”); and (3) for T*_PC/PR_*, population-level trait values are permuted within the region, preserving the number of populations in each community unchanged (null model = “regional.pop”). By accounting for the differences among communities in terms of species composition, the number of individuals, and the number of populations, we avoided the potential impact of these differences on the estimated parameters. Then, we used a direct approach to test for the significance (*p* < 0.05) of each T-statistic deriving P-values from null distributions of statistics, calculated as the proportion of the null distributions that are more extreme (on either tail) than the statistics observed values [49]. This allowed us to compare the observed statistics against the expected frequency distribution generated by null models. Besides, we quantified the magnitude of the differences between the statistics and their corresponding null models, by calculating the standardized effect size (SES) as SES = (T_obs_ − T_null_)/T_SDnull_, where T_obs_ is the observed T-statistic, and T_null_ and T_SDnull_ are the mean and the standard deviation of the null distribution, respectively [50]. The SES quantifies, in units of standard deviations, the position of the observed T-statistic within the simulated distribution. Negative SES reflects that the observed T-statistics values are lower than random expectation, whereas the opposite—positive SES scores—represents that T-statistics values are higher than random expectation. The SES has the advantage of allowing to compare the results from studies using different matrices and algorithms [51,52]. Finally, after certifying the normal distribution of T-statistics, we used ANOVAs to evaluate the potential differences between habitat types in these statistics.

#### 2.4.4. Identification of Potential External Environmental Filters Modulating Plant Community Assembly

To determine the shaping of plant assemblages through environmental filtering and to evaluate how specific lineages respond to those factors, we used a combination of the Fourth-Corner method and an extended RLQ ordination methodology [53,54]. The Fourth-Corner method was used to test for the association (*p* < 0.05) between a matrix of environmental variables (matrix R) and a matrix of plant traits (matrix Q) through randomization tests (999 permutations), using as linkage a matrix of species abundance (matrix L). The randomization test was based on a model that fixed the environmental variables and functional traits and randomized the distribution of species across the study sites [55]. This analysis avoids carrying out a large number of tests evaluating correlations between every pairwise of environmental variables and traits, which increments the chance of obtaining false significance tests. Moreover, we used the extended RLQ ordination methodology proposed by Pavoine et al. [53] to visualize the relationship between matrix R and matrix Q (including only the environmental factors significantly associated to plant traits) in a phylogenetic context (matrix P). We used the data from the species distribution and abundance across the study sites (matrix L) to link the three matrices (R, Q, and P) by weighting species and sites in ordinations by their overall relative abundance and the relative number of plants recorded, respectively. The species and site weights were derived from the canonical analysis of matrix L.

All statistical analyses were performed in R (v. 3.6.2) [56], using different packages: “phylosignal”, for phylogenetic signal tests [45]; “ape”, “corrplot” and “Hmisc” for correlation tests [57,58,59]; “vegan” for NMDS ordination and significance tests of distances among centroids [60]; “cati” for the T-statistics calculation and their corresponding null model tests [61]; and “ade4” and “adiv” for the calculation of the Fourth-Corner statistics and the extended RLQ ordination, respectively [62,63].

## 3. Results

The vegetation sampling and laboratory analysis resulted in the functional characterization of 5259 leaves from 1753 individuals representing 77 species, 58 genera, and 27 families (Table S1). The most abundant species for each community—those representing 70% of the total number of individuals—were considered in the analysis, which included, on average, 17 species for OGF and 14 species for SEF. Among these species, the most speciose families were Leguminosae (25 species), Euphorbiaceae (8 species), Annonaceae (4 species), Capparaceae (4 species), Rubiaceae (4 species), and Sapindaceae (3 species), which all together group more than 60 percent (62.34%) of the analyzed species. Moreover, the most speciose genera were *Lonchocarpus* (7 species*), Caesalpinia* (6 species), *Croton* (3 species), and *Sapranthus* (3 species), which altogether group approximately the 25 percent (24.68%) of the studied species.

Regarding the phytochemical traits, the species presented on average the following phytochemical concentrations: 30.23 mg (GAE)/100 g (range: 0.96–165.85 mg (GAE)/100 g) for total phenols; 9.92 mg (GAE)/100 g (range: 0–41.09 mg (GAE)/100 g) for total tannins and 10.07 mg (CE)/100 g (range: 0–97.37 mg (CE)/100 g) for total flavonoids (Table S1). Among the species that consistently showed the lowest concentration of total phenols, tannins and flavonoids is *Sapranthus sp*, while among the species that showed the highest levels of these phytochemicals are *Caesalpinia coriaria* for total phenols and tannins and *Acacia angustissima* for flavonoids. Regarding the other functional traits, the species presented on average the following values: 38.29 SPAD units for chlorophyll content; 170.81 cm^2^ g^−1^ for specific leaf area; 0.15 g cm^−3^ for leaf density; and 0.03 g cm^−2^ for leaf fresh mass per unit area (Table S1). On average, at the species level, we found a high variation among individuals in terms of their functional trait values. The average coefficients of variation (CVs) among species for each trait were the following (from the highest to the lowest value): 642% for LFM, 481% for LD, 312% for SLA, 259% for flavonoids concentration, 204% for tannins concentration, 182% for phenols concentration, and 88% for chlorophyll content. 

### 3.1. Relationships between Traits Related to Leaf Defense, Water Use, Light Acquisition, and Heat Load Regulation 

In general, we found a moderate level of phylogenetic signal among the studied traits, being higher for leaf density (LD), chlorophyll content (CC), and the concentration of total phenols, tannins, and flavonoids and lower for the leaf fresh mass (LFM) (Figure S1, Table S2). None of the tests for the phylogenetic signal was significant for specific leaf area (SLA).

**Figure 1:**
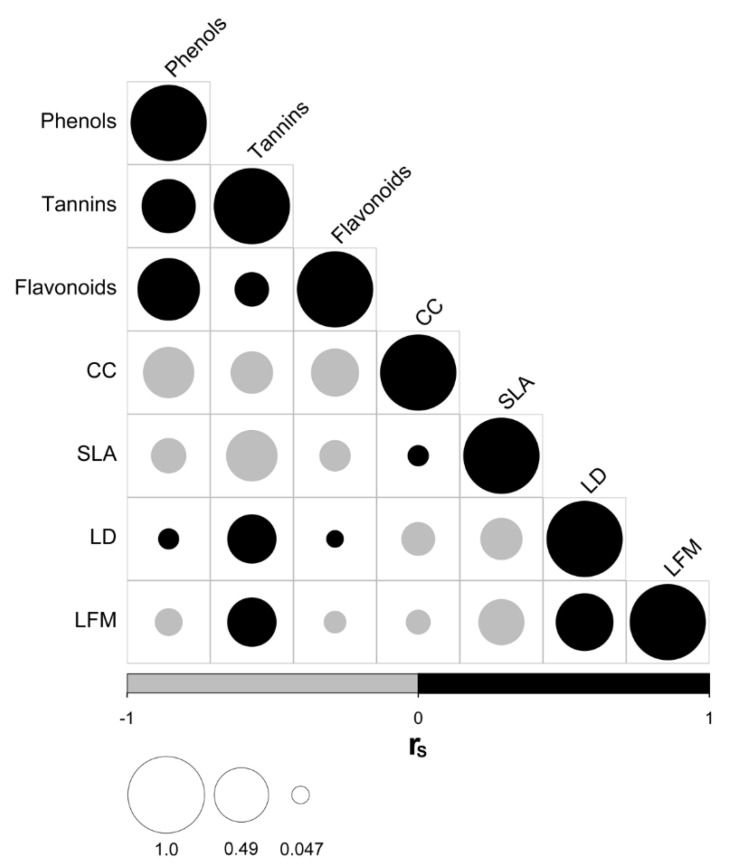
Correlograms showing relationships (r_S_: Spearman correlation coefficient) among species’ functional traits across the phylogenetic tree. Black circles indicate positive correlations, whereas gray circles indicate negative correlations. The size of the circle is proportional to the magnitude of r_S_. Traits: total phenols concentration (Phenols); tannins concentration (Tannins); flavonoids concentration (Flavonoids); chlorophyll content (CC); specific leaf area (SLA); leaf density (LD); leaf fresh mass (LFM).

Regarding the association among species traits, we found a positive correlation between total phenols, tannins, and flavonoids concentrations, which were, in turn, negatively correlated with chlorophyll content (CC), (Figure 1). Besides, we observed that the concentration of tannins was positively correlated with leaf density (LD) and leaf fresh mass (LFM), and negatively correlated with specific leaf area (SLA). These patterns were also evident in the correlations among traits at the intraspecific level (Figure S2), although the strength of the relationships substantially varies across species. The species showing the strongest relation between those traits are *Caesalpinia coriaria* and *Psidium sartorianum*, which are capable of surviving and reproducing in open areas. Finally, we also detected a positive correlation between leaf density (LD) and leaf fresh mass (LFM, both traits negatively related to SLA) across species, a pattern that is also recurrent at the intraspecific level. 

### 3.2. Contrasting OGF vs. SEF Plant Communities in Terms of Traits Composition

**Figure 2:**
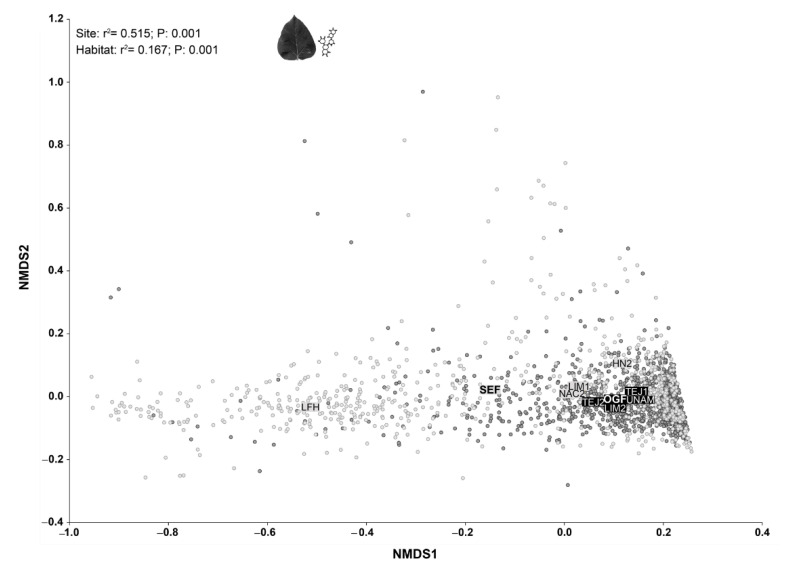
Non-Metric Multidimensional Scaling Ordinations mapping individuals’ dissimilarities in terms of their functional traits. Light gray points and labels correspond to secondary forests whereas black points and labels correspond to old-growth forests. The squares represent the centroids of the different types of habitats (secondary forest: SEF, and old-growth forest: OGF) and sampled communities (SEF: HN2, LFH, LIM1, NAC2; and OGF: LIM2, TEJ1, TEJ2, UNAM). The results of the centroids test are shown on the upper left side of the graphs.

The NMDS ordinations (Figure 2) allowed us to successfully map the individuals’ variation in terms of their functional trait values, as indicated by the low reached stress value (2.82), which according to the Kruskal’s and Clarke rules of thumb corresponds to an excellent ordination [64]. The resulting ordination showed significant differences among tree communities from the different study sites (r^2^ = 5.515; *p* = 0.001) and types of forest (r^2^ = 0.167; *p* = 0.001). On one hand, the individuals present in SEF showed, on average, higher total phenols, tannins, and flavonoids concentration than individuals in OGF: 67.36 mg (GAE)/100 g in SEF vs. 24.99 mg (GAE)/100 g in OGF for phenols concentration; 19.16 mg (GAE)/100 g in SEF vs. 8.35 mg (GAE)/100 g in OGF for tannins concentration; and 16.16 mg (GAE)/100 g in SEF vs. 9.59 mg (GAE)/100 g in OGF for flavonoids concentration. On the other hand, the individuals present in OGF showed, on average, the highest specific leaf area and leaf density: 182 cm^2^ g^−1^ in OGF vs. 142.32 cm^2^ g^−1^ in SEF for specific leaf area and 0.32 g cm^−3^ in OGF vs. 0.11 g cm^−3^ in SEF for leaf density. The observed average individual’s values for chlorophyll content (37.46 SPAD units in OGF vs. 36.19 SPAD units in SEF) and leaf fresh mass per unit area (0.03 g cm^−2^ in SEF vs. 0.02 g cm^−2^ in OGF) were quite similar in both types of forest (Table S1). 

However, as we can see in the resulting ordination (Figure 2), individuals from SEF showed greater variation in the functional traits’ values than individuals from OGF. Nevertheless, when we compared the community weighted mean values for the analyzed functional traits, we did not observe significant differences between the types of forest (Table 1), although a tendency toward more variation in SEF persists.

### 3.3. External and Internal Filters Underlying Species Coexistence in Old-Growth Forests and Secondary Forests

**Figure 3:**
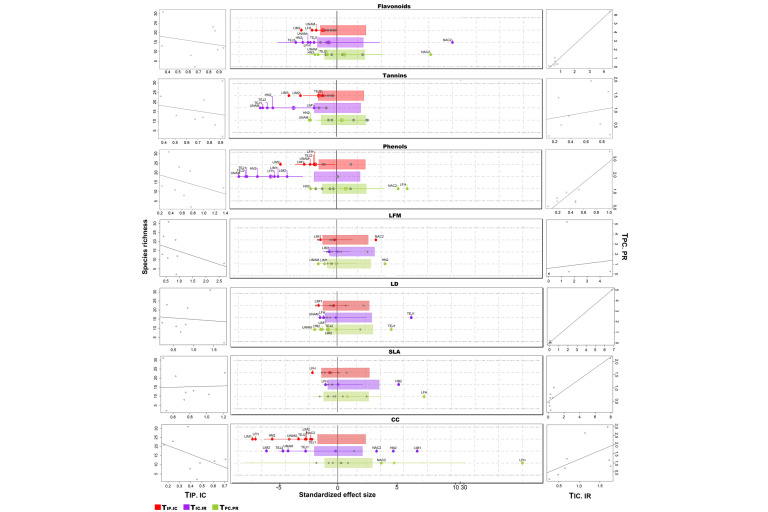
Standardized effect size (SES) of T-statistics corresponding to each tree community for the studied functional traits. Traits: total phenols concentration (Phenols); tannins concentration (Tannins); flavonoids concentration (Flavonoids); chlorophyll content (CC); specific leaf area (SLA); leaf density (LD); leaf fresh mass (LFM). T-statistics: within-population variance relative to the total variance in the community (TIP/IC); within-community variance relative to the total variance in the regional pool, assessed at the individual level (TIC/IR); and within-community variance relative to the total variance in the regional pool, assessed at the population level (TPC/PR). Dots represent the SES values for each plant community. Gray dots represent T-statistics that are not different from random expectations, whereas red, violet, and green dots represent T-statistics that do differ from random expectations (negative and positive SES indicate lower and higher values than random expectations, respectively). The crossed circles represent the SES value averaged across plant communities and the colored rectangles correspond to the average confidence intervals (0.025–0.975). Crossed circles that are not embedded within rectangles are different from random expectations. Graphs on the left show the relationship between plant community species richness and its corresponding TIP/IC, whereas graphs on the right side show the relationships between TIC/IR and TPC/PR.

The mean value of T*_IP/IC_* among communities—represented by crossed circles in Figure 3—was only different than expected by chance for total phenols concentration and chlorophyll content. The low T*_IP/IC_* values observed for those traits could be revealing nonoverlapping traits distribution among species (strong interspecific niche packing) due to a reduction in the local intraspecific variation because of the effect of internal filters. In addition, our results indicate that community-wide intraspecific variation was lower regarding total phenols concentration and chlorophyll content than regarding the other analyzed traits (flavonoids and tannins concentration, leaf fresh mass per area, leaf density, and specific leaf area) (Figure 3). In general, we observed a trend toward a decrease in the local intraspecific variation with the increase in the number of species in local communities for most traits, with the exceptions of leaf density and specific leaf area. The T*_IP/IC_* was only significantly different between habitats for chlorophyll content, being lower in secondary forest communities (Table S3).

Concerning the external filters, we found that (1) these filters may be operating with greater intensity on the phytochemical concentrations (flavonoids, tannins, and phenols), as well as on chlorophyll content—the lowest T*_IC/IR_* values were observed for those traits- (Figure 3). In fact, for total phenols and tannins concentration, the mean values of T*_IC/IR_* were significantly lower than expected by chance and filters would be operating on these traits at the individual rather than at the population level (more values are significantly lower than expected for T*_IC/IR_* than for T*_PC/PR_* and there is also a positive relationship between T*_IC/IR_* and T*_PC/PR_*). Most of the communities showing the strongest signal of potential external filtering belong to OGF and were located inside the Chamela-Cuixmala Biosphere reserve. Nevertheless, due to the high intra-habitat variation, we only observed significant differences between habitats for chlorophyll content (Figure 3, Table S3). In this case, only communities corresponding to OGF showed evidence for the potential action of external filters on its assembly process, while for the SEF communities, we observed a higher T*_IC/IR_* than expected (individuals’ variance within the community was higher than the individual variance within the regional pool). 

### 3.4. Potential Environmental Drivers of Defensive Traits Variation

The Fourth Corner test allowed us to identify a negative relationship among the phytochemical concentrations and the air average humidity (EH_avg_; r_P_ = –0.80 for total phenols concentration, r_P_ = –0.77 for total tannins concentration, and r_P_ = –0.51 for total flavonoids concentration). In addition, we found a positive association between such phytochemical concentrations and the average soil temperature (ST_avg_; r_P_ = 0.73 for total phenols concentration, r_P_ = 0.71 for total tannins concentration and r_P_ = 0.52 for total flavonoids concentration) and soil water content (SWC; r_P_ = 0.63 for total phenols concentration; r_P_ = 0.58 for total tannins concentration; and r_P_ = 0.37 for total flavonoids concentration). Finally, the Fourth Corner test also showed us a tendency toward a positive relationship between leaf density (LD), the air average temperature (ET_avg_; r_P_ = 0.36) and the photosynthetically active radiation (PAR_avg_; r_P_ = 0.18), as well as a negative relationship between these traits, the pH (r_P_ = –0.27) and the concentration of inorganic phosphorus—PO_4_^+^- available (r_P_ = –0.26). 

The first axis of the extended RLQ considering the combination of traits and environmental parameters showing some kind of relationship—identified with the Fourth Corner test—explained the 93.67% of the total variation. The phytochemical concentrations were negatively correlated with this axis (r_P_ = –0.90 for total phenols concentrations, r_P_ = –0.88 for tannins concentrations and r_P_ = –0.79 for flavonoids concentrations), as well as the following environmental parameters (ST_avg_: r_P_ = –0.93; SWC: r_P_ = –0.86; PAR: r_P_ = –0.67; and PO_4_^+^: r_P_ = –0.24). Besides, the leaf density (r_P_ = 0.18) the EH_avg_ (r_P_ = 0.73), the ET_avg_ (r_P_ = 0.33) and the pH (r_P_ = 0.28) were positively related with this axis.

**Figure 4:**
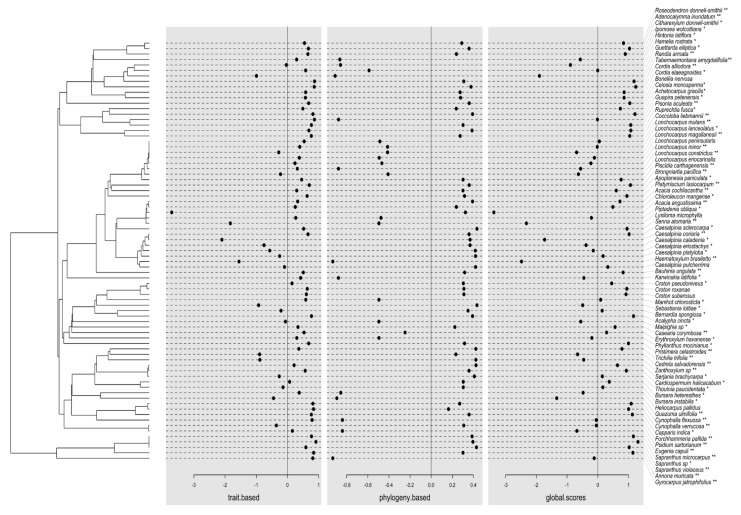
Phylogenetic tree and coordinates (dot plots) of species in the first axis of the extended RLQ analysis. The resulting coordinates (global.scores) of species were defined as a sum of a combination of traits variables (trait.based) and a combination of phylogenetic variables (phylogeny.based). * indicates species exclusively registered in old-growth forests, and ** indicates species exclusively registered in secondary forests.

These results suggest that, on one hand, the species that are more likely to be found in sites characterized by a high ST_avg_, SWC, and PAR are those with the highest concentration of total phytochemicals. Such is the case of species belonging to the families Leguminosae (v.gr. *Acacia angustissima*, *Caesalpinia caladenia*, *C. coriaria*, *C. eriostachys*, *Haematoxylum brasiletto*, *Lonchocarpus constrictus*, *L. eriocarinalis*, *L. magallanesii*, *L. minor*, *L. peninsularis*, *Lysiloma microphylla*, *Piptadenia oblicua*), Euphorbiaceae (v.gr. *Phyllantus mocinianus*, *Sebastiania lottiae*), Malvaceae, Rubiaceae, Convolvulaceae, Hernandiaceae, Malpighiaceae, Meliaceae, Myrtaceae, Rhamnaceae and Rutaceae (Figure 4). On the other hand, the species more likely to be found in the opposite environmental conditions and under a high EH_avg_, ET_avg_, and low pH, and PO_4_^+^ concentration are those with the lowest concentration of total phenols, tannins, and flavonoids that also tend to present a high leaf density (LD). Among these species are also those belonging to the families Leguminosae (v.gr. *Acacia cochliacanta*, *Apoplanesia paniculata*, *Bauhinia ungulata*, *Brongniartia pacifica*, *Caesalpinia sclerocarpa*, *Chloroleucon mangense*, *Pisonia aculeata*, *Platymiscium lasiocarpum*, *Senna atomaria*); Annonaceae, Euphorbiaceae (v.gr. *Acalypha cincta*, *Croton pseudoniveus*, *C. roxanae*, *C. suberosus*); Capparaceae, Bignoniaceae, Boraginaceae, Polygonaceae, and Sapindaceae, among others.

## 4. Discussion

Our findings revealed several important aspects of the variation of defensive phytochemicals in TDF vegetation, the effect of the successional stage on plant functional traits, and novel insights into the community assembly process in tropical communities. First, we found that the concentration of total phenols, tannins, and flavonoids varied greatly among species and individuals of the same species in most of the cases (Table S1); significant relationships among functional traits were detected when the variation at the individual level was considered. The cases with a combination of high concentrations of these compounds and a relatively low intraspecific variation were associated with abundant species capable of growing in secondary forests (i.e., *Acacia angustissima*, *Caesalpinia coriaria*), suggesting the presence of harsher biotic and abiotic (higher radiation) conditions/filters in this habitat. The opposite combination of low concentrations of phytochemicals and a relatively large intraspecific variation occurred in species associated with OGF as *Celosia monosperma,* which could be facing more favorable biotic and abiotic conditions. Other extreme cases were the species that showed very low phytochemical concentration and very low intraspecific variation such as *Annona muricata*, or the opposite pattern (very high phytochemical concentration and variation) such as *Lysiloma microphylla*. Interestingly, both species were present in abundance in SEF, suggesting that extreme strategies are also allowed in the secondary forests which coincides with the high functional variability previously registered in this habitat type [20]. Functional variability of secondary forests in TDF has been attributed to a higher environmental heterogeneity in early successional stages (i.e., regarding air temperature, relative humidity, soil temperature, vapor pressure deficit) [22] which can be associated with a higher biotic heterogeneity [65]. 

As predicted by our first hypothesis, leaf defensive traits were markedly related to other plant functional traits associated with light acquisition (CC and SLA) and leaf structure (LD and LFM). The negative relations found between the concentration of phytochemicals and chlorophyll content match the predictions of the Protein Competition Model (PCM) [66] which was proposed as the regulatory mechanism behind the carbon tradeoff assumed by the GDB Hypothesis [12]. The PCM predicts a trade-off between primary and secondary metabolisms caused by the competition between protein and phenolic compound biosynthesis for their common precursor: the phenylalanine amino acid [66]. Our detected pattern for the phenolic compounds is consistent with reports for other woody species where chlorophyll content was used as a surrogate for proteins [67,68]. A similar tradeoff between defense and photosynthesis has been found in other tropical species such as *Pentaclethra macroloba* [19] as defensive traits are costly for plants due to the energy drain from growth toward defensive metabolite production [69]. 

Additionally, a greater leaf lifespan and mechanical protection against abiotic hazards are associated with a high density of the foliar tissue and leaf toughness [70]. This explains the positive relationship between the concentration of tannins with LD and LFM, as leaf density is dependent on air spaces in the leaf tissue and, when filled with water, both density and fresh mass values increase. Leaves with this kind of structure and high tannins content are more resistant to damage by herbivores and pathogens [70]. The negative correlation of LD with SLA is not surprising; both traits are known to be negatively related, and it has been reported that an increase of LD or leaf thickness can produce a decrease in SLA [71,72]. The positive correlation of LD with mechanical and chemical defensive traits has also been reported before [73] and other studies on structural and chemical defensive traits have found positive correlations for total phenols and tannins with leaf toughness [74]. As high phytochemical content implied low CC and, especially in the case of tannins, high LD and LFM, the species with a high content of phytochemicals and associated to SEF were the ones showing the strongest relationships among these functional traits (i.e., *Caesalpinia coriaria* and *Psidium sartorianum*) (Figure S2).

It is important to note that all functional traits evaluated, except for SLA, presented a moderate phylogenetic signal which was more evident in certain families such as Leguminosae and Euphorbiaceae (Figure S1, Table S2), indicating that the most abundant clades in this TDF could share the ability to resist the harsh biotic and abiotic conditions of this tropical system by developing high levels of chemical and physical protection when exposed to conditions related to early successional stages or disturbed habitats. Indeed, habitat disturbance predisposes the vegetation to fungal infection as some important fungal pathogens are clearly adapted to the drier, warmer, brighter conditions present after forest clearance and at secondary forest and fragment edges [6,75]. In this sense, the disturbance would be altering fundamental plant functional traits/functions by exposing them to harsher biotic and abiotic conditions. Moreover, the negative association of CC with phytochemicals and LD, as well as the moderate phylogenetic signal in CC, also more evident in abundant clades, suggests that these clades could face a similar tradeoff between productive and defensive functions, especially because abundant clades suffer greater enemy pressure than rare ones and because phylogenetically clustered communities (as SEF communities with higher dominance of Legumes and Euphorbiaceae species) could experiment a greater disease/herbivory pressure as phylogenetically related species are more likely to be alternative hosts for a given pathogen/herbivore [76]. 

According to our second hypothesis, OGF and SEF plant communities showed differential patterns in their phytochemical content and variation indicating that they are under different scenarios of the GDB Hypothesis (Figure 2), possibly as a result of the anthropogenic disturbance and its consequences in biotic and abiotic conditions [6,77]. In fact, the greater functional variation of individuals from SEF indicates that species that strongly diverge along the acquisition–preservation resource axis of plant strategy (i.e., from those species with high chlorophyll content, favoring matter acquisition, to those species with high phytochemical concentrations, favoring matter preservation), coexist in stands of SEF. This could be reflecting the “storage effect” favored by the spatial environmental heterogeneity produced by the land-use history and a stochastic colonization process, which define the occurrence of plant species with contrasting attributes and consequently more heterogeneity in the environmental conditions. This could also be reflecting limits to similarity and by hence greater variation inside the abundant clades (i.e., legumes) that are more dominant in these communities (Figure 4). 

Contrary to the established paradigm for humid and rainy forest (late-successional species express higher allocation to defense) [78], we found a high concentration and variation of phytochemicals in SEF (Table S1), as in scenario 2 of the GDB Hypothesis (see above) [17]. This finding suggests that biotic and abiotic factors of this habitat favor a higher allocation to defense to protect leaves from (1) a combination of high radiation and physical damage (i.e., caused by wind and turbulence); (2) the generalist herbivores/pathogens that tend to prevail in secondary vegetation; and (3) the plant stress caused by the extreme environmental conditions, the introduction of exotic species and the changes in environmental conditions directly affecting pathogens (i.e., dew formation which favors fungal infection of leaves) [6,21,22,79]. In general, secondary tropical dry forests may favor the predominance of species with well-defended leaves, thus avoiding the positive feedback between: (1) an increase in the physiological plant stress caused by extreme environmental conditions; (2) an increase in the prevalence of infection due to pre-existing stress on plants; (3) an increase on the physiological stress caused also by pathogens; and (4) an increase on the infection due to the deteriorated physiological state of plants [6], which will prevent the establishment of plants in such harsh environments. Indeed, studies carried out in different tropical dry forest regions had corroborated the hypothesis that early successional plants exhibit leaf traits involved in the conservative use of water or evaporative cooling [20,80]. 

Conversely, the lower concentrations of phytochemicals in OGF suggest a lower requirement for photoprotection and/or chemical defense in this habitat type (but see [81]). According to the GDB Hypothesis, plant communities from this habitat (which are more phylogenetically diverse and by hence could experiment a lower disease/herbivory pressure) would represent the third scenario consisting of a low allocation of resources to defense and favored growth at higher availability of nutrients than the SEF [17,20,76].

The difference between habitats became more evident when we analyzed variation at the individual level, perhaps because interactions with the biotic and abiotic environment are ultimately based at the level of the individual, and individuals of the same species are facing different interactions depending on their specific location in a highly heterogeneous tropical system [82]. In fact, there are individual variation theories that explicitly identify intraspecific variation as the main driver of local diversity [25,83,84].

It is expected at this point that assembly processes within the studied communities would also be measurable at the individual level. As predicted in our third hypothesis, our results revealed that external filters were acting, above all, at the individual level, especially for the phytochemical concentrations. This finding indicates that from all the possible values of such phytochemical concentrations present in the regional species pool, just a limited range of these values are present in our communities (Figure 3). Provided that external filters include climate, soil, and generalist biologic agents (predators/pathogens) as processes outside the communities modulating the sorting of individuals from the regional pool [25], we consider that herbivore/pathogen pressure as well as environmental factors such as radiation, humidity, and soil conditions could filter out individuals lacking the required functional characteristics from the community. 

In OGF sites, we found, specifically, evidence for a stronger filtering effect regarding total phenols and tannins concentration (i.e., communities located within the Chamela-Cuixmala Biosphere reserve presented a stronger filtering effect than SEF communities). This lower variation in the concentration of total phenols and tannins could be due to one of the following reasons: (1) being specious communities, the OGF sites can harbor diverse communities and more specialized herbivores/pathogens, and consequently, they would experiment in greater proportion the added effect of external and internal filters [81,85]; and (2) species-rich communities can also be associated to a lower herbivore/pathogen prevalence by means of the dilution effect because, in diverse communities, most of the species, including the most effective hosts, are not highly abundant; the dilution effect could decrease the pressure to produce high concentrations of defense metabolites, allowing the assignment resources to other fundamental functions in OGF individuals [86,87]. Specifically, in our context, a dilution effect can occur in plant-pathogen and plant-herbivore systems when (1) there are marked differences between host species in terms of their quality for pathogens and herbivores, as in OGF, where different lineages of plant species with diverse functional strategies coexist; (2) pathogen and herbivores with specialized hosts and feeding habits, respectively, predominate within the community, as it may occur in OGF as a consequence of the high taxonomic and functional diversity in plant communities; and (3) plant communities are composed by a mixture of lower and higher quality hosts, which reduce the encounter rate between hosts and their specialized pathogen or herbivore [8].

Our results revealed, indeed, an internal filter regarding total phenols concentration (Figure 4). The simultaneous presence of generalist and specialist herbivores/pathogens could favor the stabilizing selection especially in specious communities [88]. This explains the decrease in intraspecific variation with the increase in species richness which reduces the overlapping among populations in total phenols values and, consequently, in their defensive strategy against generalist herbivores/pathogens. This trend toward a decreasing overlapping among populations in defensive functional traits was also observed, although with lower intensity, in the cases of tannins and flavonoids, reinforcing the idea that more specious communities (such as those of OGF) tend to present diverse defensive strategies in this tropical forest. Such diversity of species and strategies would favor a dilution effect allowing for the decrease in herbivores/pathogens prevalence as well as in phytochemicals content [77]. 

The important role that environmental conditions played in the functional performance of the studied communities was also evident in the positive association of phytochemical content with the soil temperature and the soil water content. This positive association could be the plant response to stress produced by high saturation of environmental humidity (which reached 93%), soil humidity (up to 30%), and high soil temperature (up to 30 °C). Together, these factors could generate a low availability and take-up of nutrients, possibly due to leaching or occlusion processes [89,90], especially in the case of nitrogen (TN) and phosphorus (TPho), but also, nitrogen and phosphorus concentration showed a trend toward a negative relationship with the concentration of the phytochemicals measured in our study. Our results are consistent with a study in a rain forest that detected that high concentrations of phenolic compounds in the fresh leaf litter are related to a low N availability in the soil favored by leaching processes [91]. 

As previously demonstrated by empirical studies, variation in environmental conditions and resource availability can modulate the susceptibility of plants to be attacked by herbivores and pathogens, as well as their response to such attacks [92]. Although we did not find significant differences between habitats in terms of filtering (Figure 3), we detected associations between habitats and plant phytochemical contents (Figure 2), as well as between phytochemical contents and environmental variables (Figure 4), that allowed us to elucidate differing patterns between conserved and disturbed habitats. For example, species that showed the highest phytochemical contents occurred in sites with typical environmental conditions of secondary forests (high soil temperature and radiation) [22]. Indeed, most of these species were exclusive of SEF, which reinforces the idea that higher phytochemical concentrations are being selected in habitats with harsh environmental conditions and with a greater abundance of generalist herbivores/pathogens. On the contrary, species that showed the lowest phytochemical contents also tended to present a high leaf density and occurred in sites with typical conditions of old-growth forests (low soil temperature, radiation, pH, and PO_4_^+^ concentrations). The negative relationships between leaf density, the pH, and the PO_4_^+^ concentration are also possibly a response of the vegetation to conditions of low availability of soil nutrients. We suggest that in this nutrient-limited ecosystem associated with low pH, a strategy for the protection of nutrient loss is the investment in structural metabolites in the leaf that will favor an increase in density. Our findings are consistent with another study in a seasonally dry forest which showed that the increase in soil pH produced an increase in nutrient availability, and, under these conditions, the plants tended to present high SLA and low LD [93].

Although several species with high phytochemical content belong to the family Leguminosae, the most abundant family in this TDF, and even more dominant in secondary forests [20,94], this feature is distributed in several clades along the phylogenetic tree, and the same occurs with the species with lower phytochemical concentration (Figure 4). This explains the absence of a strong phylogenetic signal in traits values as well as the detection of stronger filtering at the individual than at the population level. In this sense, intraspecific variation in phytochemical concentration would drive populations and species variation in response to the local presence of herbivores/pathogens and environmental conditions.

Concerning the implications for conservation, our findings, like others from studies evaluating the effect of anthropogenic disturbance in the vegetation functional performance (i.e., [6,20,80,85,95]), indicate that transformation of tropical dry forests, by changing biotic and abiotic conditions, alters the defensive requirements of plants and increases the need for chemical and physical defenses as well as of photoprotection. Consequently, in anthropogenic landscapes, we could expect a change in terms of ecosystem properties and services as a high concentration of total phenols has been associated with low availability of N in forest soils [96,97,98]. Indeed, forest litter with a high concentration of tannins has been related to low rates of ammonification [97] and nitrification [98] which decrease the nitrogen availability in these ecosystems; tannins in the soil could be sequestering sources of organic N, affecting the rates of nitrogen mineralization in the ecosystem [98]. In this scenario, nitrogen fixers such as legumes would have an important advantage that could underlie their dominance in disturbed forests. However, soil fertility as well as herbivore abundance and prevalence could be significantly altered with the increasingly TDF transformation [99], leading to (1) less diverse plant communities, (2) the predominance of generalist plant pathogens and herbivores which could tolerate a wide array of constitutive plant defense compounds, (3) degraded ecosystem services (i.e., lower timber production and carbon sequestration, a reduction in the aesthetic value of forest and soil quality) and (4) the provision of disservices to agricultural ecosystems (i.e., a reduction in production due to herbivory) [100]. For these reasons, anthropogenic landscapes of TDF should avoid a massive transformation that could threaten the ecosystem functioning and conservation. 

## 5. Conclusions

In conclusion, our results revealed that high phytochemical content, especially in the case of tannins, was consistently associated with high leaf density and leaf fresh mass, as thickness and toughness confer leaves resistance to drought and radiation as well as to chemical and physical defenses against herbivore and pathogen attack. The moderate phylogenetic signal found in phytochemicals and leaf density, which was more evident in the most abundant families of this TDF (Leguminosae and Euphorbiaceae), suggest that these clades could share the ability to resist the harsh biotic and abiotic conditions of this tropical system and could face a similar tradeoff between productive and defensive functions, especially because abundant clades suffer greater enemy pressure than rare ones and because phylogenetically clustered communities (as SEF communities) could experiment a greater disease/herbivory pressure as phylogenetically related species are more likely to be alternative hosts for a given pathogen/herbivore. We also found that the lower variation in phytochemical content and the stronger filtering detected in OGF sites could be the combined result of strong external and internal processes that drive community assembly in this habitat. The action of both external biotic and abiotic filters could explain the lower variation in defensive traits in OGF communities. This could also reduce the functional overlapping, allowing for the coexistence of different defensive strategies in species-rich communities where a stronger competition mediated by herbivores can occur. Additionally, greater species richness in OGF could dilute the prevalence of pathogens and herbivores. Finally, we found evidence for an important environmental filter modulating the expression of phytochemicals. For example, species that showed the highest phytochemical contents occurred in sites with typical environmental conditions of secondary forests, while species that showed the lowest phytochemical contents occurred in sites with typical conditions of old-growth forests. Our findings indicate that the transformation of tropical dry forests alters the defensive requirements of plants, increasing the need for chemical and physical defenses as well as photoprotection. Consequently, in anthropogenic landscapes, we could expect more degraded ecosystem services and even the provision of disservices to socioecosystems, as a result of the change in plant functional traits.

## Figures and Tables

**Table 1:** Community weighed mean values (CWM) regarding the study functional traits for secondary and old-growth forests.

Habitat Sites	N_Ind_	S	Phenols	Tannins	Flavonoids	CC	SLA	LD	LFM
SF									
LFH	256	2	140.47	36.52	26.14	32.30	125.47	0.13	0.02
NAC2	81	8	35.46	9.58	20.41	37.39	137.85	0.22	0.02
HN2	135	21	11.14	4.97	2.81	37.37	190.74	0.10	0.05
LIM1	238	23	31.48	11.78	11.54	39.38	149.02	0.06	0.02
Total	710	43							
Mean/SD			54.64/58.20	15.71/14.16	15.22/10.23	36.61/3.02	150.77/28.33	0.13/0.07	0.03/0.02
OGF									
TEJ1	234	12	18.97	7.36	10.30	39.29	176.94	0.54	0.02
TEJ2	244	11	31.99	9.92	13.83	36.76	174.01	0.10	0.03
LIM2	206	13	25.45	8.71	7.84	34.46	119.00	0.11	0.02
UNAM	359	31	23.89	7.73	7.23	38.44	194.59	0.09	0.02
Total	1043	42	–	–	–				
Grand total	1753	77	–	–	–				
Mean/SD			25.08/5.37	8.43/1.15	9.8/3.00	37.24/2.13	166.14/32.71	0.21/0.22	0.02/0
F/*p*-value			1.02/0.35	1.05/0.0.35	1.04/0.35	0.12/0.75	0.50/0.50	0.51/0.50	0.4/0.55

Habitat: secondary (SEF) and old-growth forest (OGF). Parameters: number of studied individuals (N_Ind_) and species (S). Functional traits: concentration of total phenol (Phenols, mg (GAE)/100 g); concentration of total tannins (Tannins, mg (GAE)/100 g); concentration of total flavonoids (Flavonoids, mg (CE/100 g); chlorophyll content (CC, SPAD unit); specific leaf area (SLA, cm^−2^ g^−1^); leaf density (LD, g cm^−3^); and leaf fresh mass (LFM, mg). The F and *p*-values are results of the ANOVA test comparing these types of forests.

## Data Availability

The data presented in this study are available in the article and Supplementary Materials.

## Supplementary Materials

The following are available online at https://www.mdpi.com/article/10.3390/plants11040516/s1, Figure S1: Visualization of traits values along the phylogenetic tree, Figure S2: Correlograms showing the relationships (r_S_: Spearman correlation coefficient) between traits for the most abundant species, Table S1: Average (±SD) concentration (mg(GAE)/100 g) of total phenols, tannins, and (mg(CE)/100 g) flavonoids; chlorophyll content (CC), specific leaf area (SLA), leaf density (LD), and leaf fresh mass per unit area (LFM) for the studied species, Table S2: Values of the metrics and its corresponding *p*-values (metric/*p*-value) used to evaluate phylogenetic signals for each studied trait, Table S3: Results of ANOVA tests (F/*p*-value) comparing T-statistics between habitat types (old-growth forest vs. secondary forest) regarding the functional traits.

## Abbreviations

Average of photosynthetically active radiatio	(PARavg)
Average soil temperatur	(STavg)
Chlorophyll conten	(CC)
Community-weighted mea	(CWM)
Diameter at breast heigh	(DBH)
Environmental/air average humidit	(EHavg)
Environmental/air average temperatur	(ETavg)
Growth-differentiation balanc	(GDB) hypothesis
Leaf densit	(LD)
Leaf fresh mas	(LFM)
Maximum sun radiatio	(SRmax)
Nitroge	(N)
Non-Metric Multidimensional Scalin	(NMDS)
Old-growth fores	(OGF)
Phosphomonoesteras	(PME)
Phosphoru	(Pho)
p-nitropheno	(pNP)
Secondary fores	(SEF)
Soil water conten	(SWC)
Specific leaf are	(SLA)
Standardized effect siz	(SES)
Total carbo	(TC)
Total flavonoid	(Flavonoids)
Total nitroge	(TN)
Total organic carbo	(TOC)
Total phenol	(Phenols)
Total phosphoru	(TPho)
Total tannin	(Tannis)
Tropical dry fores	(TDF)
T-statistic	(TIP/IC, TIC/IR, and TPC/PR)
Universal buffe	(MUB)
β-1, 4-glucosidas	(BG)
β-1, 4-N-acetylglucosaminidas	(NAG)

## Appendix A. Reagents Used for Secondary Metabolites Quantification

### Appendix A.1. Reagents

- Aluminum chloride (Sigma-Aldrich 206911)
- (+)-Catechin hydrate ((Sigma-Aldrich C1251)
- Folin and Ciocalteu’s phenol reagent (Sigma-Aldrich F9252)
- Gallic acid monohydrate (Sigma-Aldrich 398225)
- Methanol (Sigma-Aldrich 179337)
- Polyvinylpyrrolidone (PVP) (Sigma-Aldrich PVP40)
- Sodium carbonate (Sigma-Aldrich S7795)
- Sodium nitrite (Sigma-Aldrich S2252)
- Sodium hydroxide (Sigma-Aldrich S5881)
- Water deionized and distilled (dd H_2_O)

### Appendix A.2. Reagents Setup

- Aluminum chloride: Prepare a solution of 10% in methanol.
- Folin–Ciocalteu reagent: Prepare a solution of 10% (*v*/*v*) F&C in water.
- Methanol: Prepare a solution of 95% (*v*/*v*) methanol in water.
- Sodium carbonate: Prepare a solution of 700 mM Na_2_CO_3_ in water.
- Sodium hydroxide: Prepare a solution of 1 M NaOH in water.
- Sodium nitrite: Prepare a solution of 5% NaN0_2_ in water.
