# Supplementary material for: The Assembly of Tropical Dry Forest Tree Communities in Anthropogenic Landscapes: The Role of Chemical Defenses
Source: Plants (Basel). 2022 Feb 14;11(4):516. doi: 10.3390/plants11040516 (PMC8877018; doi:10.3390/plants11040516)
Supplement: Supplementary file 1 [file plants-11-00516-s001.zip › Table S3_Comparisons of T-statistics between habitats.pdf]

Table S3. Results of ANOVA tests (F / P-value) comparing *T*-statistics between habitat types (old growth forest *vs* secondary forest) regarding the functional traits.

	***T*-statistics**		
**Leaf traits**	$T_{IP/IC}$	$T_{IC/IR}$	$T_{PC/PR}$
Phenols	0.07 / 0.79	0.81 / 0.40	0.15 / 0.72
Tannins	0.01 / 0.94	0.56 / 0.48	0.56 / 0.48
Flavonoids	0.45 / 0.53	0.89 / 0.38	0.61 / 0.47
CC	***5.47 / 0.05***	**35.41 / 0**	***4.83 / 0.07***
SLA	0 / 0.10	0.89 / 0.38	0.28 / 0.61
LD	0.15 / 0.71	1.09 / 0.34	0.90 / 0.38
LFM	1.34 / 0.29	1.09 / 0.34	0.88 / 0.39

T-statistics: within-population variance relative to the total variance in the community ($T_{IP/IC}$); within-community variance relative to the total variance in the regional pool, assessed at individual level ($T_{IC/IR}$); and within-community variance relative to the total variance in the regional pool, assessed at population level ($T_{PC/PR}$). Traits: concentration (mg(GAE)/100g) of total phenols (Phenols), tannins (Tannins), and (mg(CE)/100g) flavonoids (Flavonoids); chlorophyll content (CC), specific leaf area (SLA), leaf density (LD), and leaf fresh mass per unit area (LFM). Significant values of P (< 0.05) are shown in bold, and marginal relationships (0.05 ≤ P < 1.00) are shown in bold italic.
