# Supplementary material for: The Assembly of Tropical Dry Forest Tree Communities in Anthropogenic Landscapes: The Role of Chemical Defenses
Source: Plants (Basel). 2022 Feb 14;11(4):516. doi: 10.3390/plants11040516 (PMC8877018; doi:10.3390/plants11040516)
Supplement: Supplementary file 1 [file plants-11-00516-s001.zip › Figure S1_Traits values along the phylogenetic tree.pdf]

Figure S1 Visualization of traits values (centered and scaled) along the phylogenetic tree.

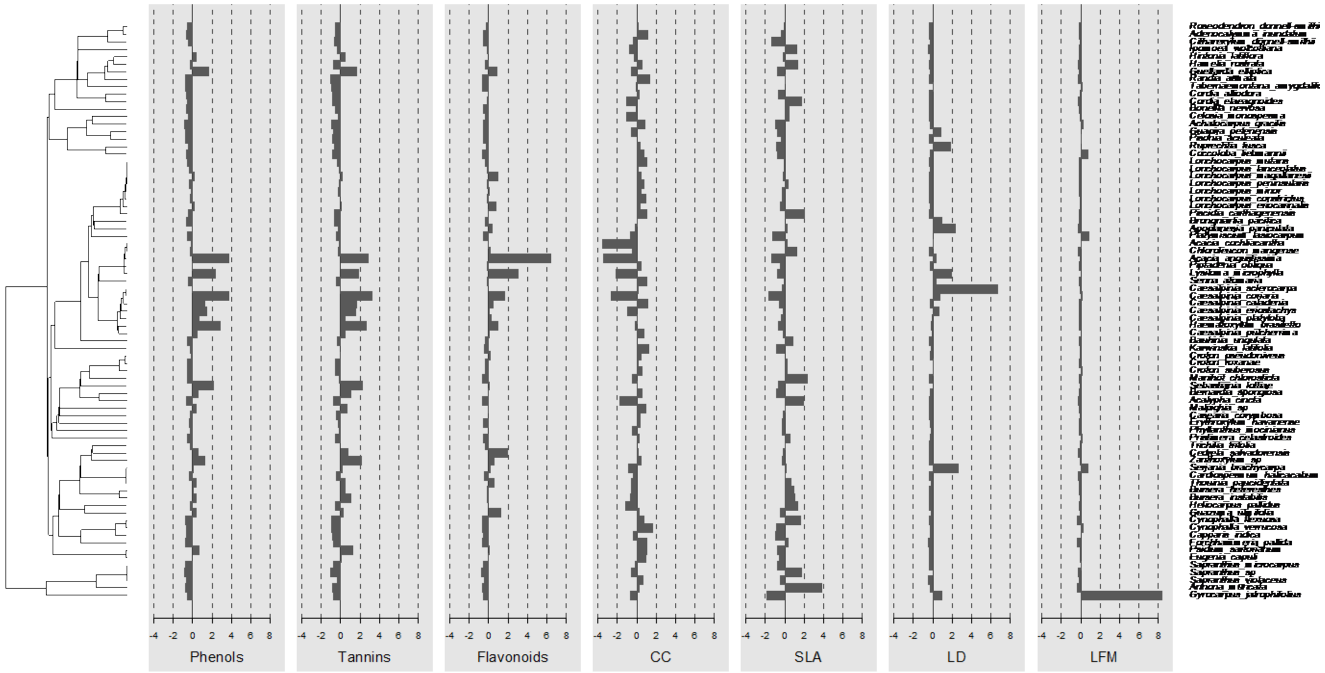

Traits: concentration (mg(GAE)/100g) of total phenols (Phenols), tannins (Tannins), and (mg(CE)/100g) flavonoids (Flavonoids); chlorophyll content (CC), specific leaf area (SLA), leaf density (LD), and leaf fresh mass per unit area (LFM).
