# Supplementary material for: The Assembly of Tropical Dry Forest Tree Communities in Anthropogenic Landscapes: The Role of Chemical Defenses
Source: Plants (Basel). 2022 Feb 14;11(4):516. doi: 10.3390/plants11040516 (PMC8877018; doi:10.3390/plants11040516)
Supplement: Supplementary file 1 [file plants-11-00516-s001.zip › Figure S2_Relationships between traits at species level.pdf]

Figure S2 Correlograms showing the relationships (r_S: Spearman correlation coefficient) between traits for the most abundant species.

LEGUMINOSAE

Acacia cochliacantha

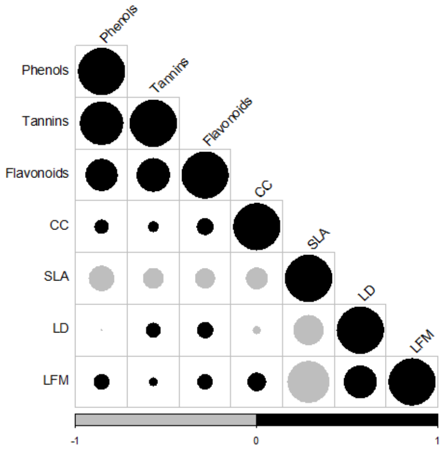

Apoplanesia paniculata

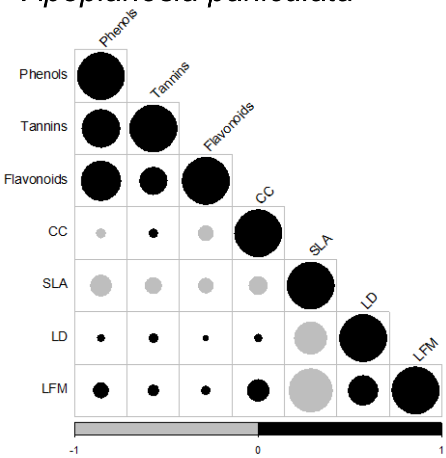

Caesalpinia coriaria

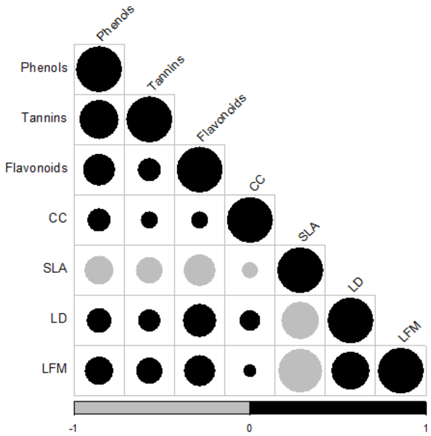

Caesalpinia eriostachys

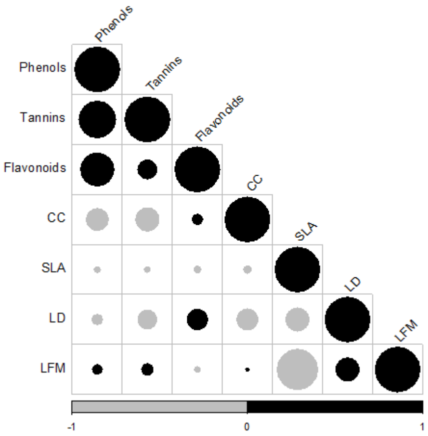

Haematoxylum brasiletto

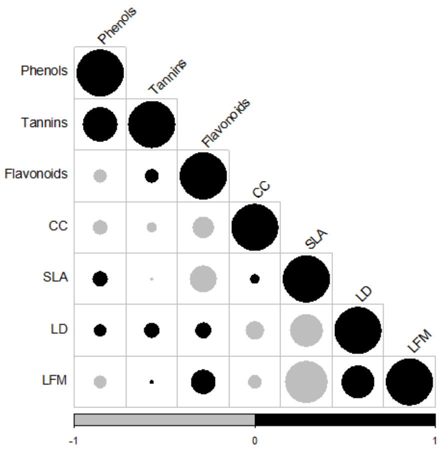

Lonchocarpus lanceolatus

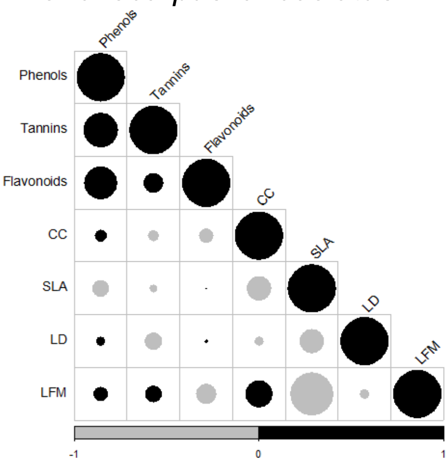

EUPHORBIACEAE

Croton roxanae

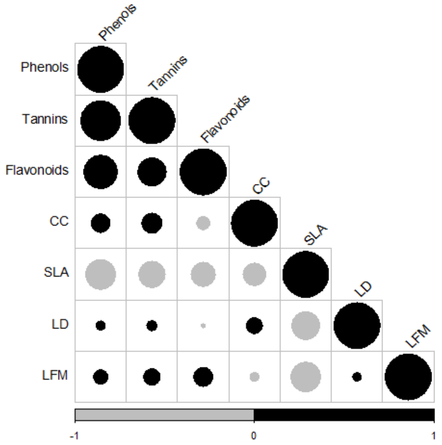

Croton suberosus

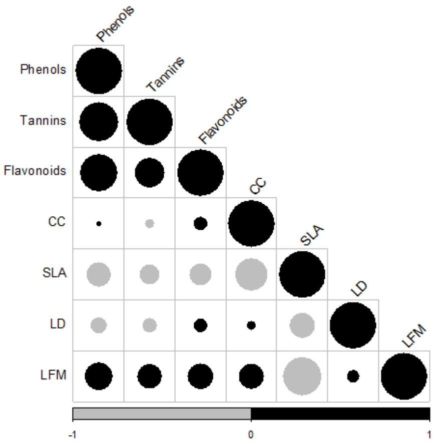

SAPINDACEAE

Serjania brachycarpa

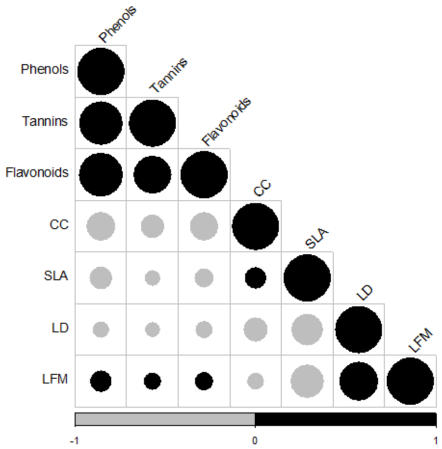

Thouinia paucidentata

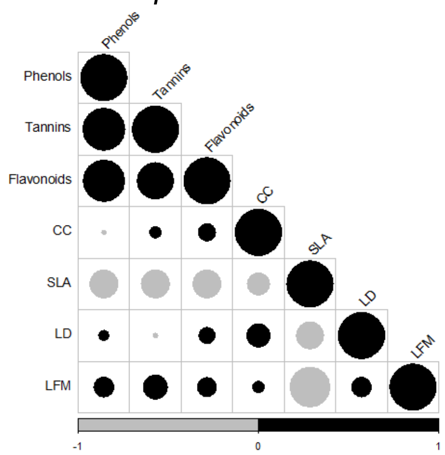

MALVACEAE

Heliocarpus pallidus

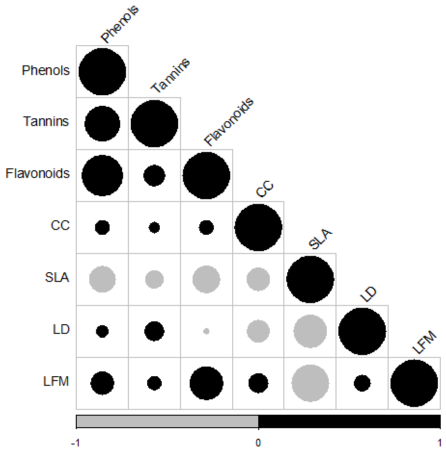

MYRTACEAE

Psidium sartorianum

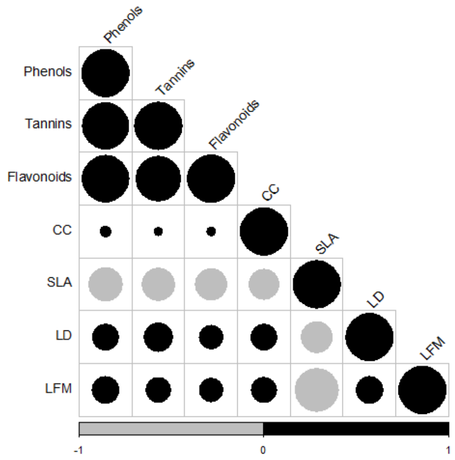

NYCTAGINACEAE
Guapira petenesis

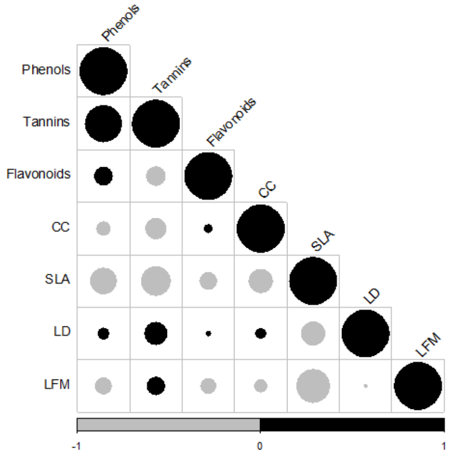

SALICACEAE
Casearia corymbosa

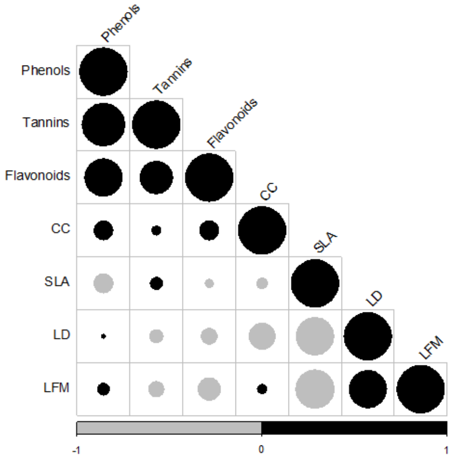

Black circles indicate positive correlations, whereas gray circles indicate negative correlations. The size of the circle is proportional to the magnitude of r_S. Traits: concentration (mg(GAE)/100g) of total phenols (Phenols), tannins (Tannins), and (mg(CE)/100g) flavonoids (Flavonoids); chlorophyll content (CC), specific leaf area (SLA), leaf density (LD), and leaf fresh mass per unit area (LFM).
