# Supplementary material for: The Assembly of Tropical Dry Forest Tree Communities in Anthropogenic Landscapes: The Role of Chemical Defenses
Source: Plants (Basel). 2022 Feb 14;11(4):516. doi: 10.3390/plants11040516 (PMC8877018; doi:10.3390/plants11040516)
Supplement: Supplementary file 1 [file plants-11-00516-s001.zip › Table S1_Phytochemical concentrations for studied species.pdf]

Table S1. Average (±SD) concentration (mg(GAE)/100g) of total phenols, tannins, and (mg(CE)/100g) flavonoids; chlorophyll content (CC), specific leaf area (SLA), leaf density (LD), and leaf fresh mass per unit area (LFM) for the studied species.

Family<br>*Species*	n	Habitat	Phenols	Tannins	Flavonoids	CC	SLA	LD	LFM
Achatocarpaceae (1)									
Achatocarpus gracilis	8	OGF	0.96±0.91	0.64±0.90	2.15±3.12	45.95±9.23	101.42±36.05	0.07±0.04	0.04±0.01
Amaranthaceae (1)									
Celosia monosperma	22	OGF	12.12±19.66	6.84±10.72	5.05±9.01	29.32±4.42	206.60±47.82	0.03±0.01	0.02±0.00
Annonaceae (4)									
Annona muricata	2	SEF	2.02±0.23	1.39±1.01	0.32±0	40.89±0.00	436.61±410.78	0.07±0.00	0.01±0.00
Sapranthus microcarpus	1	SEF	1.54	2.16	2	36.85	112.26	0.04	0.03
Sapranthus violaceus	3	SEF	6.28±2.31	5.94±1.66	3.71±1.77	44.42±3.11	132.24±16.96	0.03±0.01	0.02±0.00
Sapranthus sp	1	OGF	0.96	0	0	33.59	288.93	0.16	0.01
Apocynaceae (1)									
Tabernaemontana amygdalifolia	10	SEF	1.55±0.86	0.62±1.26	1.38±1	37.48±6.40	168.14±100.36	0.17±0.39	0.04±0.09
Bignoniaceae (2)									
Adenocalymma inundatum	4	SEF	6.33±0.74	4.29±2.91	2.49±1.52	47.89±5.21	140.38±33.76	0.05±0.04	0.02±0.01
Roseodendron donnell-smithii	4	SEF	8.74±2.80	4.75±1.25	5.97±3.35	40.10±3.35	159.77±28.50	0.03±0.01	0.02±0.00
Boraginaceae (2)									
Cordia alliodora	4	SEF	6.55±5.55	1.81±2.18	8.14±5.40	40.69±5.42	122.81±81.40	0.05±0.02	0.02±0.01
Cordia elaeagnoides	9	OGF	8.10±6.47	1.99±2.05	7.86±6.38	29.36±4.03	289.50±184.77	0.05±0.02	0.01±0.00
Burseraceae (2)									
Bursera heteresthes	1	OGF	35.28	15.10	7.27	33.61	237.36	0.05	0.01
Bursera instabilis	3	OGF	43.27±35.62	20.46±25.14	7.81±4.74	32.54±2.59	246.65±151.57	0.04±0.01	0.02±0.01
Capparaceae (4)									
Capparis indica	11	OGF	6.33±2.82	1.84±1.92	1.63±0.84	35.22±3.93	101.51±19.23	0.08±0.04	0.02±0.00
Cynophalla flexuosa	3	SEF	2.13±0.87	1.11±1.59	1.21±0.38	44.92±5.20	281.71±214.05	0.05±0.04	0.003±0.01
Cynophalla verrucosa	5	SEF	4.61±3.55	0.70±0.69	0.45±0.22	52.82±4.25	105.72±58.99	0.07±0.08	0.04±0.05
Forchhammeria pallida	3	SEF	4.13±0.85	2.33±0.57	0.35±0.31	47.77±3.18	196.32±82.53	0.03±0.01	0.004±0.00
Celastraceae (1)									
Pristimera celastroides	9	SEF	7.89±9.07	4.16±6.62	2.04±2.75	41.00±6.41	212.73±279.78	0.07±0.06	0.04±0.07
Convolvulaceae (1)									
Ipomoea wolcottiana	1	OGF	31.21	6.51	8.03	31.62	259.19	0.02	0.02
Erythroxylaceae (1)									
Erythroxylum havanense	1	OGF	16.84	7.34	2.31	41.22	147.69	0.07	0.01
Euphorbiaceae (8)									
Acalypha cincta	1	OGF	4.84	3.05	0.45	23.01	304.06	0.06	0.01
Bernardia spongiosa	1	OGF	51.85	19.83	9.21	43.68	110.51	0.08	0.02
Croton pseudoniveus	3	OGF	22.10±15.04	9.50±8.64	13.24±9.42	42.35±2.63	169.27±16.27	0.06±0.03	0.02±0.00

Croton roxanae	199	SHR	9.68±13.09	4.21±5.21	4.34±7.77	39.78±5.56	190.13±57.96	0.18±0.70	0.02±0.00
Croton suberosus	195	SHR	10.49±9.90	4.64±6.31	4.75±4.29	43.49±5.63	191.19±111.02	0.19±0.77	0.04±0.24
Manihot chlorosticta	1	OGF	10.88	8.40	0.34	33.83	329.55	0.03	0.01
Phyllanthus mocinianus	14	OGF	21.84±9.06	7.79±4.13	9.52±7.10	34.04±6.14	151.60±35.30	0.09±0.03	0.01±0.00
Sebastiania lottiae	12	OGF	108.93±46.41	31.59±18.43	10.87±3.47	39.57±5.09	118.55±17.66	0.14±0.11	0.02±0.00
Hernandiaceae (1)									
Gyrocarpus jatrophifolius	4	SEF	8.77±3.80	2.15±1.73	1.99±0.95	32.69±3.12	38.47±40.45	0.41±0.53	0.51±0.44
Leguminosae (25)									
Acacia angustissima	2	SEF	164.53±9.91	36.80±7.53	97.37±9.18	9.17±2.17	75.05±15.01	0.25±0.03	0.02±0.00
Acacia cochliacantha	46	SEF	22.46±40.89	8.49±11.62	8.41±21.81	8.30±7.30	190.83±595.88	0.17±0.20	0.03±0.03
Apoplanesia paniculata	183	OGF	25.86±11.63	7.46±4.99	15.12±7.31	36.62±4.68	187.68±63.12	0.76±3.63	0.01±0.00
Bauhinia ungulata	18	SEF	11.18±7.43	6.38±4.67	5.28±3.80	38.34±3.85	232.23±187.01	0.04±0.04	0.01±0.02
Brongniartia pacifica	26	SEF	7.52±2.50	3.50±1.92	5.05±1.04	39.18±5.24	168.20±51.45	0.40±0.38	0.02±0.00
Caesalpinia caladenia	2	OGF	75.79±38.50	26.09±9.43	17.42±10.71	48.59±4.73	129.28±11.74	0.07±0.04	0.02±0.00
Caesalpinia coriaria	54	SEF	165.85±27.57	41.09±12.61	33.55±5.86	15.38±8.83	53.57±28.03	0.36±0.44	0.04±0.01
Caesalpinia eriostachys	45	OGF	83.71±23.26	25.35±13.62	11.63±5.72	30.37±8.62	153.87±46.86	0.33±1.51	0.01±0.01
Caesalpinia platyloba	2	OGF	56.91±25.06	14.91±6.85	14.76±7.99	39.13±0.44	143.40±55.18	0.12±0.02	0.02±0.01
Caesalpinia pulcherrima	21	SHR	46.65±26.38	15.07±9.47	11.18±7.14	45.10±6.97	158.61±149.11	0.11±0.13	0.03±0.03
Caesalpinia sclerocarpa	9	OGF	27.54±39.99	8.80±12.69	10.36±13.8	39.93±7.70	152.26±53.40	1.92±5.19	0.02±0.02
Chloroleucon mangense	1	OGF	18.76	7.23	12.33	33.78	255.71	0.05	0.01
Haematoxylum brasiletto	202	SEF	133.69±34.44	35.32±14.04	24.16±10.20	36.82±4.77	120.92±31.45	0.09±0.07	0.02±0.00
Lonchocarpus constrictus	28	SEF	21.11±13.04	9.57±4.21	6.92±9.15	46.51±5.27	151.36±63.79	0.05±0.03	0.02±0.02
Lonchocarpus eriocarinalis	25	SHR	38.54±19.53	11.17±7.27	21.58±13.54	41.88±5.00	132.45±86.46	0.08±0.15	0.02±0.01
Lonchocarpus lanceolatus	51	OGF	16.91±5.52	7.81±3.93	7.01±3.22	40.37±3.85	161.12±55.16	0.07±0.04	0.02±0.00
Lonchocarpus magallanesii	11	SEF	37.66±33.40	11.76±5.56	22.91±18.61	42.14±3.82	152.59±35.58	0.04±0.01	0.01±0.00
Lonchocarpus minor	5	SEF	24.27±20.09	7.42±4.36	10.98±6.36	42.64±3.01	149.06±58.04	0.04±0.01	0.02±0.01
Lonchocarpus mutans	4	SEF	10.25±4.08	6.25±2	4.60±1.19	47.81±3.57	159.03±41.49	0.04±0.02	0.02±0.00
Lonchocarpus peninsularis	13	SHR	17.09±6.99	8.07±2.70	6.92±4.99	45.28±4.98	196.68±66.86	0.04±0.01	0.02±0.01
Lysiloma microphylla	23	SHR	115.90±80.06	27.57±18.16	51.85±34.96	20.04±13.97	114.23±66.02	0.66±1.55	0.03±0.03
Piptadenia obliqua	20	OGF	28.88±15.69	9.80±4.76	6.89±3.08	42.48±9.58	133.68±42.12	0.07±0.05	0.02±0.01
Pisonia aculeata	19	SEF	1.90±1.95	1.77±1.63	2.40±0.95	38.02±6.68	139.06±45.38	0.04±0.01	0.04±0.01
Platymiscium lasiocarpum	9	SEF	11.44±4.45	5.72±3.27	2.26±3.86	32.35±3.25	79.50±53.17	0.15±0.14	0.08±0.06
Senna atomaria	7	SEF	13.42±17.08	5.65±5.62	6.98±9.90	47.23±9.32	163.38±66.89	0.25±0.43	0.02±0.00
Malpighiaceae (1)									
Malpighia sp	1	OGF	44.38	16.58	8.43	46.56	173.05	0.04	0.02
Malvaceae (2)									
Guazuma ulmifolia	26	SEF	45.55±31.87	12.58±9.40	27.03±18.21	40.76±4.63	133.06±30.95	0.11±0.12	0.02±0.00
Heliocarpus pallidus	45	SHR	19.11±18.14	4.77±4.77	9.92±11.33	28.37±4.55	261.58±107.42	0.05±0.05	0.01±0.00
Meliaceae (2)						±			
Cedrela salvadorensis	4	SEF	52.57±11.56	17.20±7.54	37.01±11.24	40.33±3.53	154.33±19.61	0.05±0.01	0.01±0.00
Trichilia trifolia	10	SEF	20.73±20.21	10.16±9.52	4.59±5.47	38.35±10.59	176.43±57.40	0.04±0.02	0.02±0.02
Myrtaceae (2)									
Eugenia capuli	15	SEF	31.57±19.73	10.63±6.60	9.27±4.64	46.82±5.42	127.21±26.61	0.05±0.01	0.02±0.00

Psidium sartorianum	30	SEF	56.50±40.64	21.78±16.79	11.41±5.93	47.31±6.72	113.86±24.81	0.05±0.01	0.02±0.00
Nyctaginaceae (2)									
Guapira petenensis	34	OGF	2.52±2.57	2.11±2.19	1.53±1.83	33.41±4.14	113.50±36.37	0.40±1.91	0.03±0.01
Piscidia carthagenensis	7	SEF	12.24±7.67	3.86±2.13	9.40±6.34	47.59±2.86	312.29±449.59	0.03±0.01	0.02±0.01
Polygonaceae (2)									
Coccoloba liebmannii	17	SEF	7.36±1.89	1.93±1.77	1.20±1.98	45.19±3.84	112.15±107.76	0.06±0.07	0.07±0.06
Ruprechtia fusca	8	OGF	12.97±10.65	3.54±2.90	6.54±5.28	41.45±7.14	105.21±52.79	0.66±1.71	0.03±0.01
Primulaceae (1)									
Bonellia nervosa	12	SHR	6.62±4.90	4.51±5.14	1.86±1.91	37.64±5.67	205.13±165.24	0.05±0.03	0.02±0.02
Rhamnaceae (1)									
Karwinskia latifolia	2	OGF	20.77±5.77	10.20±4.60	2.84±3.75	49.23±0.88	105.32±6.72	0.10±0.02	0.02±0.00
Rubiaceae (4)									
Guettarda elliptica	1	OGF	91.51	25.88	22.66	33.41	113.93	0.06	0.02
Hamelia rostrata	2	OGF	19.37±1.08	2.20±1.46	4.54±0.13	42.95±0.00	263.94±89.18	0.04±0.01	0.01±0.00
Hintonia latiflora	4	OGF	45.30±10.22	14.21±6.67	9.67±1.65	35.64±2.45	156.72±53.78	0.06±0.01	0.01±0.00
Randia armata	1	SEF	1.82	0	0.38	50.33	178.63	0.05	0.01
Rutaceae (1)									
Zanthoxylum sp	25	SEF	74.79±32.81	30.58±16.62	17.99±8.08	42.14±11.23	146.55±54.96	0.05±0.02	0.02±0.00
Salicaceae (1)									
Casearia corymbosa	30	SEF	20.63±25.06	5.65±7.07	10.20±12.11	41.52±4.19	152.45±165.14	0.05±0.04	0.02±0.01
Sapindaceae (3)									
Cardiospermum halicacabum	1	OGF	16.38	5.29	2.88	34.78	178.99	0.05	0.01
Serjania brachycarpa	33	OGF	32.69±20.06	12.26±6.85	12.59±7.61	30.61±4.50	186.15±68.58	0.85±2.59	0.07±0.32
Thouinia paucidentata	83	OGF	45.05±22.40	14.23±9.62	18.47±11.48	33.31±3.99	217.07±114.09	0.09±0.13	0.01±0.02
Verbenaceae (1)									
Citharexylum donnell-smithii	1	OGF	13.09	3.81	2.13	35.27	75.63	0.07	0.02

n: number of sampled individuals. Habitat: old growth forest (OGF), secondary forest (SEF), and shared species (SHR).
The number of species per plant family is presented between parentheses.
