# Supplementary material for: The Assembly of Tropical Dry Forest Tree Communities in Anthropogenic Landscapes: The Role of Chemical Defenses
Source: Plants (Basel). 2022 Feb 14;11(4):516. doi: 10.3390/plants11040516 (PMC8877018; doi:10.3390/plants11040516)
Supplement: Supplementary file 1 [file plants-11-00516-s001.zip › Table S2_Phylogenetic signal for the studied traits.pdf]

Table S2. Values of the metrics and its corresponding P-values (metric / P-value) used to evaluate phylogenetic signals for each studied trait.

	**Phylogenetic Signal Metrics**				
**Traits**	**I**	**C_{mean}**	**K**	**K***	**λ**
Phenols	**0.11 / 0.01**	**0.23 / 0.01**	0.05 / 0.30	0.05 / 0.35	**0.32 / 0.01**
Tannins	**0.10 / 0.01**	**0.28 / 0**	0.06 / 0.12	0.06 / 0.16	**0.44 / 0**
Flavonoids	**0.09 / 0.03**	**0.13 / 0.04**	0.04 / 0.53	0.05 / 0.54	0.16 / 0.10
CC	**0.15 / 0.01**	0.11 / 0.07	0.03 / 0.46	0.04 / 0.47	0.19 / 1
SLA	-0.03 / 0.64	-0.14 / 0.97	0.03 / 0.32	0.04 / 0.30	0 / 1
LD	**0.06 / 0.03**	**0.11/ 0.04**	0.02 / 0.80	0.03 / 0.80	0 / 1
LFM	-0.01 / 0.39	-0.03 / 0.77	**1.04 / 0.01**	0.45 / 0.09	**0.97 / 0**

Traits: concentration (mg(GAE)/100g) of total phenols (Phenols), tannins (Tannins), and (mg(CE)/100g) flavonoids (Flavonoids); chlorophyll content (CC), specific leaf area (SLA), leaf density (LD), and leaf fresh mass per unit area (LFM). Phylogenetic signal metrics: the Moran's I (I); the Abouheif's C_{mean} (C_{mean}); Blomberg's K (K) and Blomberg's K* (K*) and Pagel's λ (λ) (Keck et al. 2016). The statistical significance ($P < 0.05$) of indexes was tested through randomization (999 permutation) for K, K* and I and by likelihood ratio test for λ.

References

Keck F, Rimet F, Bouchez A, and Franc A. 2016. Phylosignal: an R package to measure, test and explore the phylogenetic signal. Ecology and Evolution 6(9): 2774-2780.
